# Comparative Genomic Characterization of Relaxin Peptide Family in Cattle and Buffalo

**DOI:** 10.1155/2022/1581714

**Published:** 2022-10-04

**Authors:** Muhammad Saif-ur Rehman, Faiz-ul Hassan, Zia-ur Rehman, Hafiz Noubahar Hussain, Muhammad Adnan Shahid, Muhammad Mushahid, Borhan Shokrollahi

**Affiliations:** ^1^Institute of Animal and Dairy Sciences, University of Agriculture, Faisalabad 38040, Pakistan; ^2^University of Agriculture Faisalabad-Sub Campus, Toba Tek Singh 36050, Pakistan; ^3^Animal Science Division, Nuclear Institute for Agriculture and Biology (NIAB), Faisalabad, Pakistan; ^4^Department of Animal Science, Sanandaj Branch, Islamic Azad University, Sanandaj, Iran

## Abstract

Relaxin family peptides significantly regulate reproduction, nutrient metabolism, and immune response in mammals. The present study aimed to identify and characterize the relaxin family peptides in cattle and buffalo at the genome level. The genomic and proteomic sequences of cattle, buffalo, goat, sheep, horse, and camel were accessed through the NCBI database, and BLAST was performed. We identified four relaxin peptides genes (*RLN3*, *INSL3*, *INSL5*, and *INSL6*) in *Bos taurus*, whereas three relaxin genes (*RLN3*, *INSL3*, and *INSL6*) in *Bubalus bubalis*. Evolutionary analysis revealed the conserved nature of relaxin family peptides in buffalo and cattle. Physicochemical properties revealed that relaxin proteins were thermostable, hydrophilic, and basic peptides except for *INSL5* which was an acidic peptide. Three nonsynonymous mutations (two in *RLN3* at positions A16 > T and P29 > A, and one in *INSL6* at position R32 > Q) in *Bos taurus*, whereas two nonsynonymous mutations (one in *RLN3* at positions G105 > w and one in *INSL3* at position G22 > R) in *Bubalus bubalis*, were identified. *INSL3* had one indel (insertion) at position 55 in *Bos taurus*. Gene duplication analysis revealed predominantly segmental duplications (*INSL5/RLN3* and *INSL6/INSL3* gene pairs) that helped expand this gene family, whereas *Bubalus bubalis* showed primarily tandem duplication (*INSL3/RLN3*). Our study concluded that relaxin family peptides remained conserved during the evolution, and gene duplications might help to adapt and enrich specific functions like reproduction, nutrient metabolism, and immune response. Further, the nonsynonymous mutations identified potentially affect these functions in buffalo.

## 1. Introduction

The relaxin peptide family comprises seven peptides with significant structural similarities but low sequence resemblance. It includes seven genes, relaxin like *RLN1*, *-2*, and *-3*, and insulin-like *INSL3*, *-4*, *-5*, and *-6* in most mammals [1], but their number varies. These peptides show a high sequence resemblance with insulin due to the presence of six cysteine residues that provide the 2 interchain and 1 intrachain disulphide linkages. Each relaxin peptide family member is constituted of two chains called A and B chains [2]. These chains are connected by two disulfide bonds present between them and one disulfide bond within the A chain. Each chain contained the cysteine residues together with distinctive disulfide bonding, which are found conserved across all family members [2].

The *RLN1* and *RLN2* are present in humans and higher primates like apes. Both are referred to as relaxin as human RLN2 is an orthologue to RLN1 in other mammals [1, 3]. *RLN3* was first identified in 2002 and is considered the common ancestral gene for all relaxin peptides [2, 4]. *RLN1* and its orthologue *RLN2* play an important role in reproduction in mammals [5], but in bovines, these genes have been lost during the evolution and have no traces in the genome [6]. *RLN3* has been shown to play a role in nutrient metabolism in cattle [7]. *INSL3* plays a crucial role in testicular descent by promoting the growth and development of the gubernaculum ligament [8]. *INSL4* is highly expressed in the placenta and might be involved in bone development [5, 9]. Its receptor is yet to be identified. *INSL5* with its cognate receptor *RXFP4* has been suggested to play important role in immune response regulation, signal transmission to CNS through the vagus nerve, and autocrine/paracrine function within the intestinal tract [10]. *INSL6* role in sperm production has been determined in buffalo [11].

Relaxin family peptides have important role in ruminant reproduction and nutrient metabolism as mentioned above. The availability of genomic data has provided opportunity to perform genome-wide characterization of protein families using the different available bioinformatics tools. Many studies have been conducted to identify the evolutionary relationships, physicochemical characteristics, comparative amino acid analysis, effects of mutations, and gene duplications in important protein families in ruminants [12–15]. The present study was conducted to characterize relaxin family peptides in cattle (*Bos taurus*) and buffalo (*Bubalus bubalis*) at genome-wide level in order to better understand their evolutionary significance, physicochemical properties, and gene duplications events.

## 2. Materials and Methods

### 2.1. Genome-Wide Identification of Relaxin Peptides

The whole genome and proteomic sequence data of buffalo (UOA WB 1), cattle (ARS-UCD1.2), sheep (Oar rambouillet v1.0), goat (ARS1), camel (CamDro3), and horse (EquCab3.0) were obtained from the National Center for Biotechnology Information (NCBI) database [16]. A genome-wide BLAST and HMM search was performed to look for all putative relaxin peptide genes in *Bos taurus*, *Bubalus bubalis*, and other targeted species [17]. The cattle (*Bos taurus*), buffalo (*Bubalus bubalis*), goat (*Capra hircus*), sheep (*Ovis aries*), horse (*Equus caballus*), and camel (*Camelus dromedarius*) relaxin peptide sequences were also validated through the UniProt database search [18]. All accession numbers of sequences used for this study are presented in Table S1. No information was available for *INSL5* gene annotation of *Bubalus bubalis* in the databases.

### 2.2. Phylogenetic Analysis

Relaxin peptide amino acid sequences from *Bos taurus*, *Bubalus bubalis*, *Capra hircus*, *Ovis aries*, Equus caballus, and *Camelus dromedarius* were aligned in ClustalW. Further, the neighbor-joining (NJ) tree was constructed through the MEGA7 software [19].

### 2.3. Gene Structure, Motif Patterns, and Conserved Domain Analysis of Relaxin Peptide Family

The conserved motifs in the dataset were analyzed using the MEME suite [20]. As a query, the relaxin protein sequences were submitted in Fasta format, and a site distribution with one occurrence of each site was determined for each sequence. The motifs' minimum and maximum widths were found to be 6 and 50, respectively. The number of themes was limited to ten. The Gene Structure Display Server (GSDS) [21] was used to import all CDs and genomic sequences. The final gene structure was exhibited and illustrated using the genome annotation data in general feature format utilizing in-house scripts in the TBtools software (GFF).

### 2.4. Physicochemical Properties of Relaxin Proteins

The online available ProtParam tool [22] was used to evaluate the physicochemical characteristics of relaxin peptides including molecular weight (MW), amino acid count (AA), isoelectric point (pI), and aliphatic index (A.I.). Besides, it also included instability index (II) and the grand average of hydropathicity (GRAVY).

### 2.5. Multiple Sequence Analysis (MSA)

Multiple align show [23] was used online to explore the mutations and indels in the relaxin peptides using the aligned sequences of *Bos taurus*, *Bubalus bubalis*, *Capra hircus*, and *Ovis aries*.

### 2.6. Mutational Analysis

Further, the mutations found in *Bos taurus* and *Bubalus bubalis* relaxin peptide sequences were subsequently examined using several online tools (PolyPhen-2, MUpro, PROVEAN, IMutant, PhD-SNP, SIFT, SNAP2, PredictSNP, Meta-SNP, and SNAP) to determine their effects on protein structure and functions.

### 2.7. Nuclear Hormone Receptor Sites Identification

The NHR scan [24] was employed to predict nuclear hormone receptor binding sites. Using genomic sequences in Fasta format, NHR scan was performed. The cumulative probability of entering match states was 0.01 using the NHR scan.

### 2.8. Synteny Analysis and Gene Duplications

To find collinear genes, the whole genomes of cattle and buffalo were blasted to each other. The dual-synteny map was constructed using the TBtools after submitting annotation files for both genomes, including information about collinear genes and chromosomal IDs.

Chromosomal locations of relaxin genes were obtained from genomic resources of respective specie. An annotated genome file was saved as a general feature format (GFF) file and was fed into the MCScanX programme [25], which was subsequently used to plot the gene locations on chromosomes and presented in TB tools. In addition, the relaxin peptide gene collinearity plots for *Bos taurus* and *Bubalus bubalis* were generated. Further, for the *Bos taurus* and *Bubalus bubalis* relaxin peptide gene family, pairwise alignment of homologous gene pairs of relaxin peptide genes using MEGA7 [19] with the MUSCLE method was utilized to analyze the frequency of duplications. DnaSP v6.0 [26] was also used to determine pairwise synonymous substitutions per synonymous site (ks) and nonsynonymous substitutions per nonsynonymous site (ka) that were corrected for multiple hits. Synonymous mutations are referred as silent mutatins which can result in altered DNA sequence but does not change the encoded amino acid (evolutionary neutral mutations), whereas nonsynonymous mutations cause change in both DNA and protein sequence (evolutionary important mutations).

## 3. Results

### 3.1. Phylogenetic Analysis

**Figure 1:**
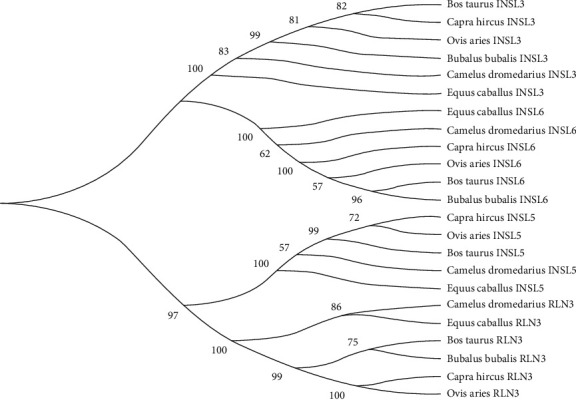
Evolutionary relationship of relaxin peptides (*RLN3*, *INSL3*, *INSL5*, *INSL6*) in six mammalian species.

Results revealed that relaxin peptide family consisted of four genes (*RLN3*, *INSL3*, *INSL5*, and *INSL6*) in all representative species, except for *Bubalus bubalis* which had 3 of them excluding *INSL5* (Figure 1). All four genes were clustered into two sister clades. From top to downward, clade 1 included *INSL3* and *INSL6*, whereas clade 2 included *INSL5* and *RLN3*. Overall phylogenetic analysis of the relaxin peptide family revealed that the “*Bos taurus* and *Bubalus bubalis*,” “*Capra hircus* and *Ovis aries*,” and “*Camelus dromedarius* and *Equus caballus*” had close similarities between them. However, *INSL3* showed more evolutionary similarities between “*Bos taurus* and *Capra hircus*” than between “*Bos taurus* and *Bubalus bubalis*.”

### 3.2. Structural Categorization of the Relaxin Peptide Family

**Figure 2:**
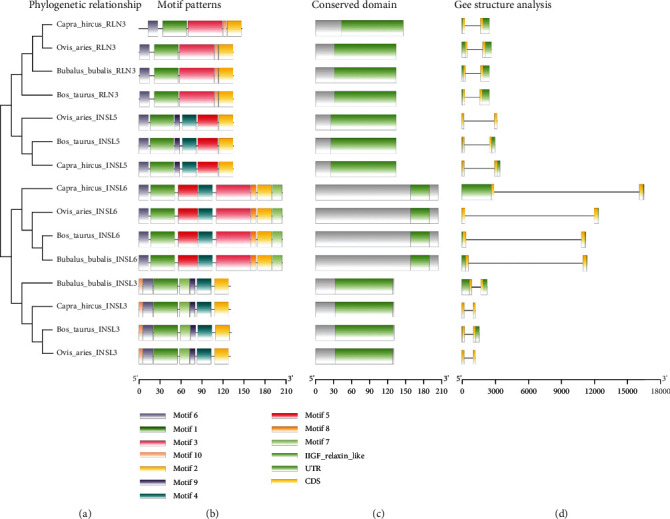
Graphical representation of motif patterns (b), conserved domains (c), and gene structure (d) of relaxin peptide family genes corresponding to their phylogenetic relationships (a) in four mammalian species.

**Figure 3:**
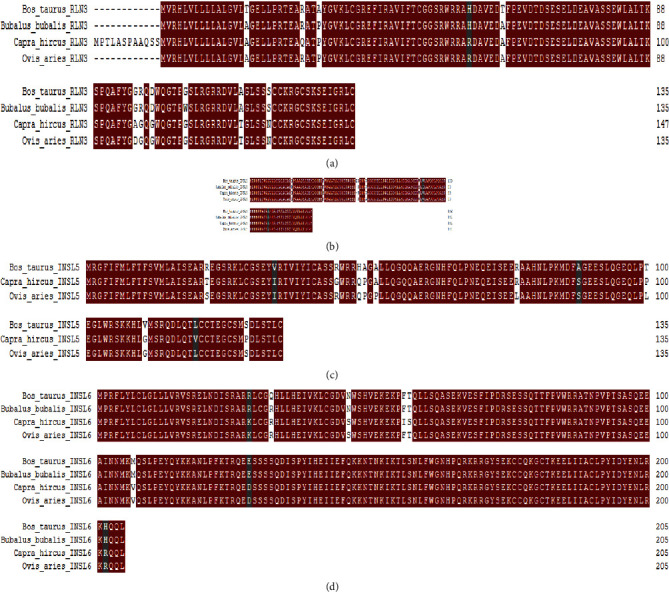
(a–d) Comparative amino acid analysis of the relaxin peptide family(*RLN3*, *INSL3*, *INSL5*, and *INSL6*) in *Bos taurus*, *Bubalus bubalis*, *Capra hircus*, and *Ovis aries.*

The examination of gene organization, motif patterns, and conserved domains in the relaxin peptide family of four targeted species, including *Bos taurus*, *Bubalus bubalis*, *Capra hircus*, and *Ovis aries*, were performed to carry out the structural characterization of the relaxin peptide family while taking account of their evolutionary relationships (Figures 2(a)–2(d)). The top ten MEME-conserved motifs were investigated to look for conserved domains (Table 1). No conserved domain was detected in the Pfam search for these motifs. Further, all genes were investigated to look for conserved domains across all targeted species (Figure 3(c)). The insulin/insulin-like growth factor (IIGF) domain was found conserved across all targeted species, which was further validated through a conserved domain database (CDD) BLAST. A gene structural analysis revealed the evolutionarily conserved nature of relaxin family genes across the studied species (Figure 3(d)). The same gene across different species revealed a similar number of introns and exons.

### 3.3. Physico-chemical Properties of the Relaxin Proteins

The physicochemical properties like location on the chromosome, exon count, molecular weight (Da), number of amino acids (A.A) in each peptide, aliphatic index (A.I.), isoelectric point (pI), instability index (II), and grand average of hydropathicity index (GRAVY) of the relaxin peptides were evaluated in cattle through the ProtParam tool (Table 2). The molecular weight of relaxin peptides ranged from 14 to 24 kDa. *RLN3* and *INSL3* were located on chromosomes 7, whereas *INSL5* and *INSL6* were located on chromosomes 3 and 8, respectively. All relaxin peptide genes had an exons count of 2 and a variable length of the peptides with amino acid residues. The pI values revealed all relaxin peptides were basic except for INSL5 which was slightly acidic. The AI values suggested the thermostable nature of all peptides having AI values greater than 65. Moreover, II values greater than 40 revealed that all peptide members of the relaxin family are unstable in vitro. Negative GRAVY values suggested the hydrophilic nature of relaxin peptides.

### 3.4. Identification of Mutations in Relaxin Peptides

Comparative amino acid analysis was performed by aligning the protein sequences of the relaxin peptides of buffalo, cattle, goat, sheep, camel, and horse in multiple align show to look for indels and single amino acid variations in *Bos taurus* and *Bubalus bubalis* (Figures 3–3(d)).

In *RLN3* protein, mutations were observed in *Bos taurus* at positions A16 > T, P29 > A, and A62 > T and *Bubalus bubalis* at position G105 > W (Figure 3). *INSL3* had one indel (insertion) at position 55 in *Bos taurus* (Figure 3(b)). In *INSL3*, mutations were observed in *Bubalus bubalis* at positions G22 > R, V86 > M, and V88 > I, whereas no mutation was observed in *Bos taurus* (Figure 3(b)). *INSL5* sequence of *Bubalus bubalis* was not found in the database. *INSL6* had a mutation in *Bos taurus* at position R32 > Q, whereas no mutation was detected in *Bubalus bubalis*.

Additionally, the mutations observed in *Bos taurus* and *Bubalus bubalis*through a comparative amino acid analysis were further analyzed through different online available mutational analysis tools to predict the functional effects of these mutations (Table S2). In *Bos taurus*, a total of three nonsynonymous mutations were predicted, two in *RLN3* at positions A16 > T and P29 > A, and one in *INSL6* at position R32 > Q. In *Bubalus bubalis*, a total of two nonsynonymous mutations were predicted, one in *RLN3* at positions G105 > w, G22 > R, and one in *INSL3* at position G22 > R.

### 3.5. NHR Patterns in Relaxin Peptides

**Figure 4:**
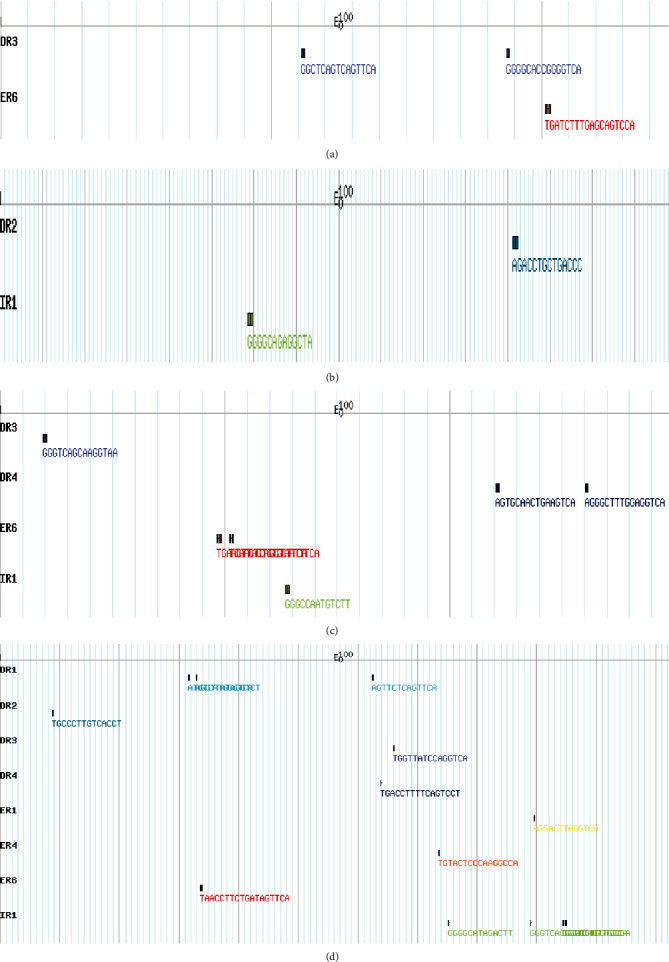
NHR scans patterns of *RLN3* (a), *INSL3* (b), *INSL5* (c), and *INSL6* (d) in *Bos taurus.*

The *Bos taurus* nuclear hormone receptor sites (NHRs) were searched for all four relaxin peptides (*RLN3*, *INSL3*, *INSL5*, and *INSL6*) (Figures 4(a)–4(d)). A total of 23 NHRs were found of which *RLN3* had 3, *INSL3* had 2, *INSL5* had 6, and *INSL6* had 12. The number of direct repeats (DR) identified in *RLN3*, *INSL3*, *INSL5*, and *INSL6* were 2, 1, 3, and 6, respectively. The number of everted repeats (ER) identified in *RLN3, INSL3, INSL5,* and *INSL6* were 1, 0, 2, and 3, respectively. The number of inverted repeats (IR) identified in *RLN3*, *INSL3*, *INSL5*, and *INSL6* were 0, 1, 1, and 3, respectively.

### 3.6. Synteny Analysis and Gene Duplications

**Figure 5:**
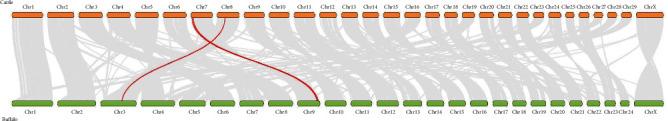
Synteny plot between *Bos taurus* and *Bubalus bubalis* genomes.

Collinearity analysis showed that relaxin family genes were randomly distributed over 2 chromosomes in both cattle and buffalo (Figure 5). In *Bos taurus*, relaxin peptide genes were present on chromosomes 7 and 8, whereas in *Bubalus bubalis*, these genes were located on chromosomes 3 and 9.

Further, the gene duplication analysis was performed to look for segmental or tandem duplication gene pairs in the relaxin peptide family of *Bos taurus* and *Bubalus bubalis* (Table 3). In *Bos taurus*, two segmental duplication events were observed between *INSL5/RLN3* and *INSL6/INSL3* gene pairs, whereas in *Bubalus bubalis*, one tandem duplication was detected between *INSL3/RNL3* gene pair. The number of nonsynonymous substitutions per nonsynonymous site/number of synonymous substitutions per synonymous site (ka/ks) ratios was determined for this duplicated event. *Bos taurus* segmental duplicated pairs *INSL5/RLN3* and *INSL6/INSL3* showed 0.68 and 0.60 ka/ks ratios, respectively, whereas *Bubalus bubalis* tandem duplication pair *INSL3/RNL3* showed 0.74 ka/ks ratio.

## 4. Discussion

### 4.1. Phylogenetic Analysis

In recent years, genomic sequencing technology, particularly next-generation sequencing, has advanced significantly, resulting in the accessibility of sequenced genomes for many important organisms, opening up a new path for understanding the genomic architecture at the molecular level of diverse animal species [27]. Comparative genomics allows for the discovery of new genes and functional components [28, 29]. Advances in bioinformatics have enabled the utilization of genomic data and look into the protein family evolutionary history, comparative amino acid analysis, gene duplications and prediction of mutations, and their functional and structural effects [12–15].

The relaxin peptide family has been found to contain seven members in most mammals, including relaxin-like genes *RLN1*, *RLN2*, and *RLN3* and insulin-like genes *INSL3*, *INSL4*, *INSL5*, and *INSL6* [30]. In our analysis, we have found four genes (*RNL3*, *INSL3*, *INSL5*, and *INSL6*) in *Bos taurus*, *Capra hircus*, *Ovis aries*, *Camelus dromedarius,* and *Equus caballus*, whereas three genes (*RNL3*, *INSL3*, and *INSL6*) in *Bubalus bubalis* from the sequenced genome of these species. Further, these genes were grouped into two sister major clades, clade 1 included *INSL3* and *INSL6*, whereas clade 2 included *INSL5* and *RLN3*. A variable number of *RLN/INSL* peptides were also observed in different vertebrates [31–33]. These variations could be explained on the basis of gene loss and fixation during the evolution and adaption to specific niches. Overall phylogenetic analysis of the relaxin peptide family revealed that the “*Bos taurus* and *Bubalus bubalis*,”, “*Capra hircus* and *Ovis aries*,” and “*Camelus dromedarius* and *Equus caballus*” had close similarities between them. Previous studies also revealed evolutionary similarities between these species [12, 13]. However, *INSL3* peptide showed more evolutionary similarity between “*Bos taurus* and *Capra hircus*” than between “*Bos taurus* and *Bubalus bubalis*”. Researchers have been fascinated by relaxin evolution for decades. Relaxins are renowned for their high sequence variability across closely related species, although unexpected parallels have been found between quite different species like pigs and whales [34].

### 4.2. Structural Features of Relaxin Peptides

The examination of gene organization, motif patterns, and conserved domains of the relaxin peptide family of four targeted species including *Bos taurus*, *Bubalus bubalis*, *Capra hircus*, and *Ovis aries* revealed the conserved nature of relaxin genes across the targeted species. The insulin/insulin-like growth factor (IIGF) domain was found conserved in all relaxin family genes across all targeted species. The insulin/IGF system (IIGFs) regulates a wide range of physiological processes, including development, linear growth, and aging, as well as metabolism, homeostasis, and central nervous system activities [35, 36]. This domain is important for proper function, and any dysregulation in this domain can result in abnormal growth, increased development and progression of numerous cancers, and pathologic ailments associated with chronic inflammation and fibrosis [37, 38].

### 4.3. Physico-chemical Properties of Relaxin Peptides

The physicochemical properties of the relaxin peptide family proteins were evaluated in *Bos taurus* through the ProtParam tool (Table 2). The molecular weight of relaxin peptides ranged from 14 to 24 kDa. The aliphatic index (AI) tells about the thermostability of globular proteins, and values greater than 65 show greater thermostability [39]. In our study, all relaxin family peptides were found thermostable. In vitro stability of proteins can be inferred through the instability index (II), and the II value lower than 40 indicates the in vitro stability of proteins [40], as in our case, all relaxin family peptides showed in vitro instability having values greater than 40. The GRAVY values tell about the hydropathicity of protein, the negative GRAVY values show hydrophilic nature, whereas the positive GRAVY values show hydrophobic nature of proteins [41], as in our case, all relaxin family peptides showed hydrophilic nature having negative GRAVY values.

### 4.4. Comparative Mutational Analysis

Comparative genomics is a large-scale, integrated technology for the comparison of two or more genomes. Comparative studies at various levels of the genomes may be conducted to obtain distinct perspectives on the organisms [35, 42]. We aligned the sequences of four species *Bos taurus*, *Bubalus bubalis*, *Capra hircus*, and *Ovis aries* in multiple align show to look for indels and single amino acid variations in *Bos taurus* and *Bubalus bubalis*. All relaxin family peptides were found well conserved with few amino acid variations in *Bos taurus* and *Bubalus bubalis*. *INSL3* had one indel (insertion) at position 55 in *Bos taurus*. Indels and mutations have all played a part in the divergence of gene family members from their progenitors [43]. Further, the mutational analysis of observed single amino acid variations predicted three nonsynonymous mutations (two in *RLN3* at positions A16 > T and P29 > A and one in *INSL6* at position R32 > Q) in *Bos taurus*, whereas two nonsynonymous mutations (one in *RLN3* at positions G105 > w, G22 > R, and one in *INSL3* at position G22 > R) in *Bubalus bubalis*. *RLN3* gene was observed to play role in feed efficiency in cattle [7]. *INSL3* is a gender-specific gene that is produced in Leydig cells of male adult and fetus and plays a key role in testicular descent [44]. Higher level of *INSL3* gene was observed in female ruminant blood with male fetus. Mutations in the *INSL3* gene resulted in failure of testicular descent (cryptorchidism) [45, 46]*. INSL6* was detected to play a role during spermatogenesis [11]. The deficiency of *INL6* in mice resulted in a decline in sperm production and immotility [47]. Mutations in these genes can interfere with functions like feed metabolism, testicular descent, and spermatogenesis in bovines.

### 4.5. NHR Patterns in Relaxin Peptides

Diverse biochemical mechanisms are involved in gene regulation and information flow from the DNA to the protein that is transcription and translation, and understanding of these mechanisms is necessary to explore the cell dynamics [48]. Nuclear receptors bind to target genes at sites referred as hormone response elements (HREs) and help to regulate the transcription. These HREs are usually located in the 5-flanking region of target genes. Even though HREs are primarily found near the primary promoter, they can sometimes be found several kilobases upstream away from the start of the transcription site in enhancer regions [49]. Most of the time, a single NHR has been found to impact many genes, and sometimes, many NHRs have competition for one target gene and result in overlapping networks for the target genes [50]. This competition for the same target gene sometimes results in reduced expression of the gene. The expression of the gene can also be reduced if NHR bind with negative HREs [49]. The pattern of NHR sites in the relaxin peptide family in *Bos taurus* was investigated. A total of 23 NHR sites were detected. In total, 12 direct repeats (DR), 6 everted repeats (ER), and 5 inverted repeats (IR) were found in the *Bos taurus* relaxin genes.

### 4.6. Synteny Analysis and Gene Duplications

Chromosomal regions common between two genomes with the same homologous genes order as in common ancestor sites are called synteny blocks [51]. Different species originating from the common ancestor in the same tree of life can be compared using syntenic relationships, which will give an idea about the chromosomal structure and number variation between species [52, 53]. Synteny analysis revealed that relaxin peptide genes were randomly located over 2 chromosomes in both *Bos taurus* and *Bubalus bubalis*. In *Bos taurus*, relaxin genes were present on chromosomes 7 and 8, whereas in *Bubalus bubalis*, these genes were located on chromosomes 3 and 9. Further, the gene duplication events were examined for *Bos taurus* and *Bubalus bubalis*. Gene duplications have evolutionary significance as it is believed that during the evolution, whole genome duplications occurred and only 5 to 10% of duplicated genomes got fixed to perform specific functions, while others were lost in the process [54, 55]. These duplication events helped in the expansion of genome size and increased complexities to perform specific functions as indicated by two rounds of duplications hypothesis (2R hypothesis) [56, 57]. In our study, the *Bos taurus* relaxin peptide family showed predominantly segmental duplications (*INSL5/RLN3* and *INSL6/INSL3* gene pairs) that helped in the expansion of this gene family, whereas *Bubalus bubalis* showed predominantly tandem duplication (*INSL3/RLN3*). Our results are in agreement with Liu et al. [58], and they explained that segmental duplications are predominant in cattle genome and these duplications in bovine genomes are enriched with specific biological processes related to digestion, lactation, immunity, and reproduction. Further, the ka/ks ratios were lower than 1 for all these observed duplications, indicating the purifying pressure for these duplication events [59].

## 5. Conclusions

Our study revealed four relaxin peptide family genes (*RLN3*, *INSL3*, *INSL5*, and *INSL6*) in *Bos taurus*, whereas three relaxin peptide genes (*RLN3*, *INSL3*, and *INSL6)* in *Bubalus bubalis* in contrast to seven genes in most of the mammals. The loss of genes might be the result of adaptation to specific niches during evolution. Relaxin family peptides remained conserved during evolution. Nonsynonymous mutations in *RLN3*, *INSL3*, and *INLS6* can interfere with biological functions like spermatogenesis, testicular descent, and feed metabolism in bovines. The segmental duplication in *Bos taurus* and the tandem duplication in *Bubalus bubalis* of relaxin family peptides helped in enrichment to specific functions like reproduction and feed metabolism during evolution.

## Figures and Tables

**Table 1:** Top ten differentially conserved motifs detected in relaxin peptide family (*RLN3*, *INSL3*, *INSL5*, and *INSL6*).

Motif	Protein sequence	Length	Pfam domain
MEME-1	REAAATEAARKLCGRHFIRAVVKLCGGSRWSREEG	35	—
MEME-2	RGLSEKCCKKGCTKSELLTLC	21	—
MEME-3	PEYQYPEVBLPFESELEEAVASSEILPLTKEPIEFYGKNTBKIGTPSNLF	50	—
MEME-4	DPALNPAPQPLSQEEAIHNMK	21	—
MEME-5	TQLLSZASEKVESFIPDRSESSQTTFPVW	29	—
MEME-6	MRALVLLLLALAVLL	15	—
MEME-7	LPGGDYELLRKLZGL	15	—
MEME-8	WGNHPQRK	8	—
MEME-9	HLLHGLMA	8	—
MEME-10	GDRDPL	6	—

**Table 2:** Physicochemical properties of the relaxin family peptides in *Bos taurus.*

Gene	Chromosome	Exon count	MW (kDa)	A.A	pI	AI	II	GRAVY
*RLN3*	7	2	14.75	135	7.60	86.00	55.82	−0.162
*INSL3*	7	2	14.38	132	8.69	96.21	67.33	−0.145
*INSL5*	3	2	15.36	135	6.89	73.04	76.93	−0.389
*INSL6*	8	2	24.00	205	9.14	77.51	66.54	−0.717

MW: molecular weight; A.A: number of amino acids; pI: isoelectric point; AI: aliphatic index; II: instability index; GRAVY: grand average of hydropathicity index.

**Table 3:** Analysis of duplicated gene pairs and their ka/ks values of relaxin peptide family in *Bos taurus* and *Bubalus bubalis.*

*Bos taurus* Gene pair	Chromosome	Duplication	ka	ks	ka/ks
INSL5/RLN3	3/7	SD	0.32	0.47	0.68
INSL6/INSL3	8/7	SD	0.33	0.56	0.60

*Bubalus bubalis* Gene pair	Chromosome	Duplication	ka	ks	ka/ks
INSL3/RLN3	9/9	TD	0.37	0.50	0.74

ka: number of nonsynonymous substitutions per nonsynonymous site; ks: number of synonymous substitutions per synonymous site; SD: segmental duplication; TD: tandem duplication.

## Data Availability

All data are shown within the manuscript.

## Supplementary Materials

Supplementary Materials Table S1: Relaxin peptides gene family members' nucleotide and protein sequences accessions from NCBI database (Genebank accessions) for *Bos taurus*, *Bubalus bubalis*, *Capra hircus, Ovis aries*, *Camelus dromedarius* and *Equus caballus*. Table S2: Mutational effects predicted through different online software in relaxin peptides of *Bos taurus* and *Bubalus bubalis.*
