# Supplementary material for: Comparative Genomic Characterization of Relaxin Peptide Family in Cattle and Buffalo
Source: Biomed Res Int. 2022 Oct 4;2022:1581714. doi: 10.1155/2022/1581714 (PMC9553489; doi:10.1155/2022/1581714)
Supplement: Supplementary Materials — Table S1: Relaxin peptides gene family members' nucleotide and protein sequences accessions from NCBI database (Genebank accessions) for Bos taurus, Bubalus bubalis, Capra hircus, Ovis aries, Camelus dromedarius and Equus caballus. Table S2: Mutational effects predicted through different online software in relaxin peptides of Bos taurus and Bubalus bubalis. [file 1581714.f1.docx]

**Supplementary table 1 (S1).** Relaxin peptides gene family members’ nucleotide and protein sequences accessions from NCBI database (Genebank accessions) for *Bos taurus*, *Bubalus bubalis*, *Capra hircus* *Ovis aries*, *Camelus dromedarius* and *Equus caballus. (Camelus dromedarius* and *Equus caballus* only protein accession number as they were used for only phylogenetic analysis)

| **Specie** | **Protein** | **Nucleotide accession** | **Protein accession** |
| --- | --- | --- | --- |
| **Bos taurus (cattle)** | RLN3 | NC_037334.1 (11667676-11665208) | XP_002688796.1 |
|  | INSL3 | NC_037334.1 (5325752-5327331) | O77801 |
|  | INSL5 | NC_037330.1 (78520247-78523226) | NP_001193060.1 |
|  | INSL6 | NC_037335.1 (39351712-39362936) | Q32L79 |
| **Bubalus bubalis (buffalo)** | RLN3 | NC_037553.1 (99441820-99444297) | XP_025149456.1 |
|  | INSL3 | NC_037553.1 (105018861-105016607) | XP_006057978.2 |
|  | INSL6 | NC_037547.1 (102229527-102240898) | XP_025137332.1 |
| **Capra hircus (goat)** | RLN3 | NC_030814.1 (97056047-97053582) | XP_005682316.2 |
|  | INSL3 | NC_030814.1 (103470021-103468808) | NP_001272508.1 |
|  | INSL5 | NC_030810.1 (42402829-42399363) | XP_005678352.2 |
|  | INSL6 | NC_030815.1 (39210866-39227365) | XP_005683754.1 |
| **Ovis aries (sheep)** | RLN3 | NC_040256.1 (10006655-10003974) | XP_004009298.1 |
|  | INSL3 | NC_040256.1 (5327208-5328420) | Q4TUB8 |
|  | INSL5 | NC_040252.1 (44666507-44663336) | XP_004002105.2 |
|  | INSL6 | NC_040253.1 (78937335-78924935) | XP_004004408.1 |
| **Equus caballus (horse)** | RLN3 | NC_009150.3 (46104607-46107745) | XP_023501009.1 |
|  | INSL3 | NC_009164.3 (3280413..3281556) | XP_023481226.1 |
|  | INSL5 | NC_009148.3 (91207719..91217200) | XP_001500253.1 |
|  | INSL6 | NC_009166.3 (26017628-26036440) | XP_001917260.3 |
| **Camelus dromedarius (camel)** | RLN3 | NC_044532.1 (9261890-9286642) | XP_010992138.2 |
|  | INSL3 | NC_044532.1 (11928576-11930216) | XP_010993407.2 |
|  | INSL5 | NC_044523.1 (51716430-51719611) | XP_010987303.1 |
|  | INSL6 | NC_044514.1 (19047444-19054999) | XP_010993544.2 |

**Supplementary table 2 (S2).** Mutational effects predicted through different online softwares in relaxin peptides of *Bos taurus* and *Bubalus bubalis*

|  | **Mutation** | **Polyphen2** | **Mupro** | **I-Mutant** | **Phd-Snp** | **SIFT** | **SNAP^2^** | **Predict SNP** | **Meta SNP** | **Overall Effect** |
| --- | --- | --- | --- | --- | --- | --- | --- | --- | --- | --- |
| **INSL3** | | | | | | | | | | |
| 1 | **G22R**  **Buffalo** | POSSIBLY  DAMAGING | INCREASE | Decrease | Neutral | Not-Tolerated | Effect | Neutral | Neutral | Non-Synonymous |
| 2 | **V86M**  **Buffalo** | BENIGN | DECREASE | Decrease | Neutral | Tolerated | Neutral | Neutral | Neutral | Synonymous |
| 3 | **V88I**  **Buffalo** | BENIGN | DECREASE | Decrease | Neutral | Tolerated | Neutral | Neutral | Neutral | Synonymous |
| **INSL6** | | | | | | | | | | |
| 1 | **R32Q**  **Cow** | PROBABLY  DAMAGING | DECREASE | Decrease | Neutral | Not-Tolerated | Effect | Deleterious | Neutral | Non-Synonymous |
| **RLN3** | | | | | | | | | | |
| 1 | **A16T**  **Cow** | UNKNOWN | DECREASE | Decrease | Neutral | Tolerated | Effect | Neutral | Neutral | Non-Synonymous |
| 2 | **P29A**  **Cow** | PROBABLY  DAMAGING | DECREASE | Decrease | Neutral | Tolerated | Effect | Neutral | Neutral | Non-Synonymous |
| 3 | **A62T**  **Cow** | BENIGN | DECREASE | Decrease | Neutral | Tolerated | Neutral | Neutral | Neutral | Synonymous |
| 4 | **G105W**  **Buffalo** | PROBABLY  DAMAGING | INCREASE | Decrease | Neutral | Not-Tolerated | Effect | Neutral | Neutral | Non-Synonymous |
